# The Generalized Analytical Expression for the Resonance Frequencies of Plasmonic Nanoresonators Composed of Folded Rectangular Geometries

**DOI:** 10.1038/s41598-018-37275-2

**Published:** 2019-01-10

**Authors:** Hai Lu, Lijun Li, Jun Zhang, Shiqiang Xia, Xiubao Kang, Meng Huang, Kesheng Shen, Chao Dong, Xianzhou Zhang

**Affiliations:** 1 Engineering Laboratory for Optoelectronic Technology and Advanced Manufacturing, Henan Normal University, Xinxiang, 453007 China; 2 Key Laboratory of Advanced Micro-structured Materials, Ministry of Education, School of Physics Science and Engineering, Tongji University, Shanghai, 200092 China

## Abstract

A robust generalized analytical expression for resonance frequencies of plasmonic nanoresonators, which consists of folded rectangular structures, is proposed based on a circuit route. The formulation is rigorously derived from the lumped circuit analogue of the plasmon resonance in a rectangular metallic nanorod. Induced by the nonhomogeneous charge distributions in the plasmonic resonators of rectangular end-caps, the electromagnetic forces drive the harmonic oscillations of free electrons in the plasmonic nanoresonators, generating intrinsically nonlinear shape-dependent LC resonance responses. Even for the plasmonic nanoresonators with much larger structure sizes than the skin depths, the significant frequency deviations due to the phase-retardation behavior can still be adequately described by the generalized expression. Moreover, for a large range of plasmonic nanoresonators with various folded rectangular geometries, sizes and materials, the generalized analytical expression gives the underlining physics and provides accurate predictions, which are perfectly verified by a series of numerical simulations. Our studies not only offer quantitative insights of nearly any plasmonic nanoresonators based on folded rectangular geometries, but also reveal potential applications to design complex plasmonic systems, such as periodic arrays with embedded rectangular nanoresonators.

## Introduction

Plasmonic nanoresonators are related to the subwavelength confinements of electromagnetic fields, and open up a wide range of applications in spectroscopy^1^, bio-sensing^2^, imaging^3^ and information technology^4^. Although there are a rich variety of plasmonic nanoresonators, they can be differentiated based on their geometries. For example, compared with spherical metallic resonators, the plasmonic nanoresonator with rectangular geometry has a longitudinal plasmon resonance, which is particularly desirable for practical applications and fabricating with versatile tools, such as electron-beam lithography (EBL)^5–10^. Moreover, since the electromagnetic field is highly localized in the closed circular geometry and the decay length of the field is limited (*δ*_*Ag*_ < 35 nm for silver), very small gaps (<20 nm) are required to achieve any noticeable coupling between the resonators with circular geometries^11–13^. In reality, though structures with lateral dimensions as small as 30 nm can be routinely generated using EBL, to achieve such a request is still a challenge^14–16^. In contrast to the plasmonic resonators with closed circular geometries, the efficient coupling distance between the plasmonic nanoresonators with rectangular geometries can be up to ~220 nm^7^. Thus it may be more suitable for use as constituent element of complex plasmonic structure^17–20^.

In order to understand and design the functionality of highly integrated devices utilizing various plasmonic nanoresonators with rectangular geometries, it is mandatory to know the exact resonance frequencies of the individual constituents, that is, the folded plasmonic rectangular nanoresonators. Usually, to determine resonance frequencies of various plasmonic systems, numerous attempts are focused on numerical techniques, such as finite element method and finite difference time domain method^21–24^. However, because of the trial-and-error nature of the numerical technique, designing often is time-consuming and the numerical results cannot be extended to other structures. Thus, to deeply exploit the resonance effects of plasmonic nanoresonators with rectangular geometries, a generalized analytical description of the interaction between light and nanoresonator is urgently needed, which can be implemented to predict directly resonance frequencies of various rectangular nanoresonators.

Plasmon resonance is an optical phenomenon arising from the collective oscillation of conduction electrons in a metal when the electrons are disturbed from their equilibrium positions^25,26^. For a bulk metal of infinite sizes in all three dimensions, the resonance frequency can be described by *ω*_*p*_ = (*Nq*^2^*/ε*_0_*m*_*e*_)^1/2^, where *N* is the number density of conduction electrons, *ε*_0_ is the dielectric constant of vacuum, *q* is the charge of an electron, and *m*_*e*_ is the effective mass of an electron^27,28^. Thus, the bulk plasmon frequency of a particular metal depends only on its free electron density. In practice, the controllable resonance frequency can be obtained by designing metallic structures of finite sizes. Specially, for a metal particle, the electron’s oscillation is subject to the boundary conditions relying on the nanoparticle’s geometry. Thus, the resonance frequency of the plasmonic nanoresonator is sensitive to resonator shape, which determines how the free electrons are polarized and distributed on the surface. Also for this reason, various shapes of metal nanoresonators (such as gold colloid) appear colorful in liquid or glass^29,30^. Nowadays, it has been known that, the resonance frequencies can be modulated in many ways by tailoring the sizes, shapes, and environments of plasmonic resonantors. For example, a flat metal–vacuum interface boundary condition results in a surface plasmon mode of *ω*_*p*_/$\sqrt{2}$ in frequency^31^, whereas for a spherical plasmonic resonator, the resonance frequency of the conduction electrons would be changed to *ω*_*p*_/$\sqrt{3}$^32^.

Because of their high symmetries, simple structures such as isolated small plasmonic spheres exhibit resonance frequencies which can be forecasted analytically. However, for more complicated shapes with lower symmetries, such as cubes or bars, a close-form solution cannot be reached easily. Inspiringly, some universal formulas for predicting resonance frequency shifts Δ*f* of any type of plasmonic nanoresonators were reported^33,34^. But they cannot determine the resonant frequency *f*_0_ analytically. Moreover, to evaluated frequency shifts Δ*f*, the scattered field or the field of resonance mode needs to be calculated firstly with extra numerical solvers according to these approaches, which compromises the efficiency of this method. Currently, another promising alternative is to treat rectangular nanoplasmonic system as a high frequency circuit composed of capacitors, inductors, and resistors^35–39^. However, most of them are limited to specific rectangular geometries or requirements, such as split-ring resonators^35,36^, plasmonic plate^37^, nanorod with large height-diameter aspect ratio^38^, or extracting the circuit parameters assisted by numerical simulations^39^. To our best knowledge, a generalized analytical prediction for the resonance frequencies *f*_0_ of plasmonic resonators with arbitrary rectangular geometries is still missing in the literature.

In this work, for the first time, a generalized closed-form expression for the resonance frequency of a single nanoresonator with arbitary rectangular cross section is derived. After that, based on a detailed analysis of the spectral positions of the resonances as a function of the nanoresonator geometry and by simulating the near-field, we further outline the fundamental difference between the present work and the previous theoretical works of the same kind, i.e. the Faraday inductance and phase retardation will play an important role when the size of rectangular cross section are large enough. Finally, we test the closed-form expression validity and universality for plasmonic resonators composed of different folded rectangular geometries with different sizes, and surrounding mediums. This work represents a study of unprecedented coherence, as we are able to correlate the resonance frequency with geometry of the individual plasmonic resonantors and a simple circuit model.

## Circuit Equations for the Plasmonic Resonators With Rectangular Geometry

**Figure 1:**
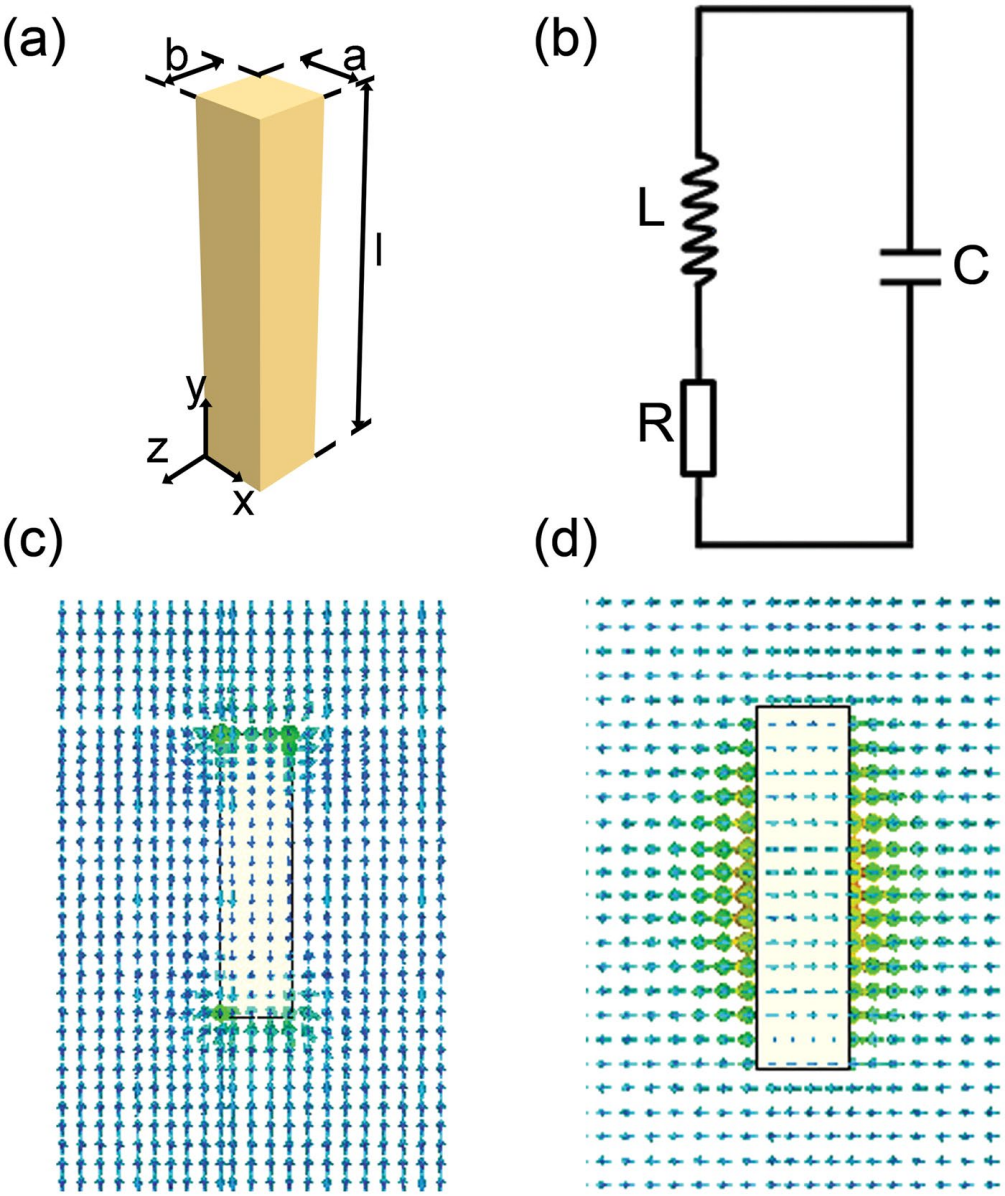
(**a**) Schematic view of the rectangular plasmonic nanorod embedded in air, and the incident light is propagating with the electric field along the rod axis, thus exciting the longitudinal plasmon resonance. (**b**) Effective circuit mapping of plasmon resonance of rectangular nanorod; inductance and resistance stand for metal resonator, capacitor represents surrounding space. (**c**) The electric field and (**d**) magnetic field distribution which located at z = −b/2 plane when a current flow in the rod is excited by the light electric field at 375 THz. In the simulation, *a* = *b* = 30 nm, *l* = 160 nm, and a Drude model *ε*_*m*_ = 1−*ω*^2^_*p*_/(*ω*^2^−i*ω*Γ) is adopted for the relative permittivity of silver with *ω*_*p*_ = 7.25 × 10^4^ cm^−1^ and Γ = 1.45 × 10^2^ cm^−1^.

A typical rectangular plasmonic nanorod and all relevant geometrical parameters are shown in Fig. 1a. The parameters in the present work are chosen to match closely those of technologically feasible physical systems^5–10^. The resonator is made of silver and assumed to be surrounded by dielectric medium with a refractive index of n_b_. In the present work, the dielectric functions of silver as published by M. A. Ordal *et al*. are used^40^. The incident light propagates along the -z axis with the electric field along the rod axis (y axis), thus exciting the longitudinal plasmon resonance. Since the metal permittivity is negative in the optical frequency region and is inversely proportional to the frequency squared, we can model a metal resonator as an inductance *L*. The interaction of a metal resonator with electromagnetic field can be then presented as excitation of *R*-*L*-*C* contour, which is depicted in Fig. 1b. Here, inductance *L* (with small losses described by resistance R) represents the metal resonator while capacitance *C* represents the surrounding space. The resonance in *R*-*L*-*C* contour is analogous to the longitudinal charge oscillations along a single nanorod. The equation of motion for this circuit is analogous to that of a driven mass on a damped spring(e.g., a Lorentz model for an atom). In general, all three circuit components must be included in lumped optical circuit models for plasmonic resonators, capacitance, kinetic inductance (*L*_*k*_), and Faraday inductance (*L*_*f*_)^37^. Intuitively, the electric potential energy is captured by a capacitor *C*; the electron kinetic energy and the magnetic field energy are captured by a kinetic inductor *L*_*k*_ and a Faraday inductor *L*_*f*_, respectively. Thus the circuit has a resonance with natural frequency ${f}_{0}=1/2\pi \sqrt{LC}$, where the inductance *L* equals the sum of the *L*_*k*_ and *L*_*f*_. It should be mentioned that, the minimum size of the nanoresonator considered in this work should be much larger than the Fermi wavelength (~0.5 nm), so that the quantum effect can be ignored and the above-mentioned classic description of the plasmonic resonance effect is applicable.

### Kinetic Inductance

Unlike the flat metallic surface plasmon whose effective kinetic inductance *L*_*k*_ needs to refer to the exact despersion relation of the surface plasmon to get the analytical expression^37^, the charge oscillation in the plasmonic nanoresonators are localized and its effective *L*_*k*_ can be obatined approximatively according to the usual expression for the bulk plasmons. Under the driving of a time-harmonic field with the angular frequency *ω*, the velocity of electrons and the current density cannot be in phase with the driving field due to the presence of electron inertia. As derived in Supporting Information I, the kinetic inductance *L*_*k*_ can be expressed as

1 $${L}_{k}={\mu }_{0}l{c}^{2}/({\omega }_{p}^{2}s)$$

where *ω*_*p*_ = (*nq*^2^/*mε*_0_)^1/2^ is the bulk plasma frequency, *q* is the electric charge, *s* = *a* × *b* is the cross-section area of rectangular nanorod, *ε*_0_ and *μ*_0_ are permittivity and permeability of vacuum, respectively, and *c* is the light velocity.

### Faraday Inductance

As can be seen in Fig. 1c,d, under the incident light, a current flow *I* will be generated in the nanorod, which is accompanied by a magnetic field with the amplitude proportional to the current. To obtain effective Faraday inductance *L*_*f*_ of plasmonic nanorods, the usual method is to calculate the self-inductance which is induced by the magnetic field energy stored both inside and outside the conductor^38^. This method assumes that the magnetic energy in the internal part of nanorod is much smaller than the external part, thus a simplified expression for *L*_*f*_ can be obtained. However, when the size of cross section is large enough, the internal part of the magnetic energy is comparable to the external part. In that case, this method will no longer accurately predict the resonance frequency. Therefore, we cannot neglect the inside magnetic field energy in the calculations. Fortunately, the self-inductance of a nanorod can be regarded as a special case of the mutual inductance between two conductors^41^. We can think of the self-inductance of a conductor as the mutual inductance between two identical conductors which coincide with each other. As derived in Supporting Information II, with the help of Neumann’s formula for calculating the mutual inductance between two parallel conductors, we can get the effective Faraday inductance *L*_*f*_ of plasmonic nanorod with rectangular cross section as

2 $${L}_{f}=\frac{\mu l}{2\pi }(\mathrm{ln}\,\frac{\sqrt{{l}^{2}+{g}^{2}}+l}{g}-\frac{\sqrt{{l}^{2}+{g}^{2}}}{l}+\frac{g}{l})$$

where *g* is the geometric-mean-distance of the parallel conductors. Note that, considering the neglected quantum effects and the physics of *L*_*f*_ related to magnetic energy, the width of the nanorod (*a* or *b*) should be much larger than the Fermi wavelength for ensuring the validity of eq. 2. The further discussion on this limitation can be found in Supporting Information II.

### Effective Capacitance

As a consequence of the current flow, electric charges with different signs will accumulate on the opposite ends of the nanorod. Correspondingly, the two end-caps of the nanorod will function as one rectangular capacitor. With the potential and charge in these capacitors, the effective capacitance along the longitudinal direction (*y* axis) can be determined. Nonetheless, it should be mentioned that the potential of a rectangluar end-cap is not homogeneous, and the two dimensional charge distribution in the end-cap of a nanorod cannot be considered as a cosine function which has been used for a single metal surface^37^. For the capacitance of the complex structures, various approaches such as conformal mapping are widely adopted to obtain quantitatively accurate closed form. However, the potential of the rectangular plasmonic disk is clear and can be worked out analytically with $V={\int }_{-a/2}^{a/2}{\int }_{-b/2}^{b/2}\sigma (x,z)dxdz/(4\pi {\varepsilon }_{b}\sqrt{{x}^{2}+{z}^{2}})$, where the *ε*_b_ is the permittivity of the surrounding medium. The effective capacitance is defined as the electric charges divided by the potential difference between the two disk centers, as derived in Supporting Information III, then we can obtained the expression for the effective capacitance *C* as follow:

3 $$C=\frac{ab\pi {\varepsilon }_{b}}{b\,\mathrm{ln}\,\frac{\sqrt{{a}^{2}+{b}^{2}}+a}{b}+a\,\mathrm{ln}\,\frac{b+\sqrt{{a}^{2}+{b}^{2}}}{a}}$$

### The Amendment of Capacitance and Faraday Inductance

In our analytical model, the effective capacitance *C* will be modified by the effects such as the inhomogeneous distribution of the potential and the weak coupling between the two disks. Besides, the Faraday inductance *L*_*f*_ will be modified also by the non-rotational symmetry of the two conductors used for calculating the mutual inductance. To take account of these effects, we phenomenologically introduce correction factors *α* and *β* to the effective capacitance and Faraday inductance, respectively. And the final expression for resonance frequency can be rewritten as:

4 $${f}_{0}=\frac{1}{2\pi \sqrt{\alpha C({L}_{k}+\beta {L}_{f})}}$$

As can be seen in eq. 4, in order to understand and even design the functionality of highly integrated devices utilizing plasmonic resonators with rectangular geometry, it is mandatory to exactly know the properties of individual units, especially the correction factors. Here, the correction factors can be determined by comparing the analytically and numerically obtained resonance peaks. For the silver rectangular resonators studied in this paper, an appropriate value of *α* = 2.65 and *β* = 0.65 have been found from the calculation about rectangular nanorod and will be used in related resonators with folded geometries. It should be mentioned that, although the correction factors *α* and *β* are artificially introduced, these parameters are instructive and can be obtained easily through experimental results of the simplest structure (i.e. nanorod) according to the user’s respective process conditions^8^. Moreover, the correction factors can also be directly applied to other geometries folded from the rectangular nanorod with the same constituents. A thorough discussion is provided in the Supporting Information IV.

## The Effect of the Faraday Inductance and Phase Retardation

### Subwavelength Rectangular Plasmonic Nanorods

In the radio frequency and microwave regimes, the resonance frequency *f*_0_ of a resoantor is only a function of characteristic lengths *l*, and in certain conditions a linear relationship between them can be resulted. For example, an ideal half-wave dipole resonator is made of a thin rod of length *l* = *c*/2*f*_0_. Particularly, at optical frequencies, for a plasmonic nanorod with large aspect ratio (*l*/*r* ≫ 1), a linear relationship can be found between the absorption maximum of the longitudinal plasmon resonance and the mean aspect ratio or the medium dielectric constant^42^. Other than these special cases, our approach demonstrates a more general nonlinear relationship between the resonance frequency and the geometry parameters of the subwavelenght rectangular plasmonic nanorod as shown in the eq. 4. In order to test the developed theory we numerically analyzed the subwavelength rectangular plasmonic nanorod with different geometrical parameters. Numerical simulation are carried out by a finite integration technique solver (CST microwave studio), with Drude-type dispersion of silver considered^40^. Here, our simulations are performed in the frequency domain with unit cell boundary to simulate a nanorod arrays, which is a straightforward and reliable approach. When a plane wave incident these nanorod arrays, the resonate frequency *f*_0_ can be obtained directly via monitoring the characteristic transmission spectra. The periods in both x and y directions are set to 700 nm which is far greater than the propagation distance of the evanescent field to weaken the coupling effects between adjacent particles. The objective of this paper is to analyze the impact of geometry on the resonate frequency of the optical nanoresonator more accurately. For this purpose, we use adaptive tetrahedral mesh refinement for higher accuracy and radiation boundary conditions to truncate the unbounded domain.

**Figure 2:**
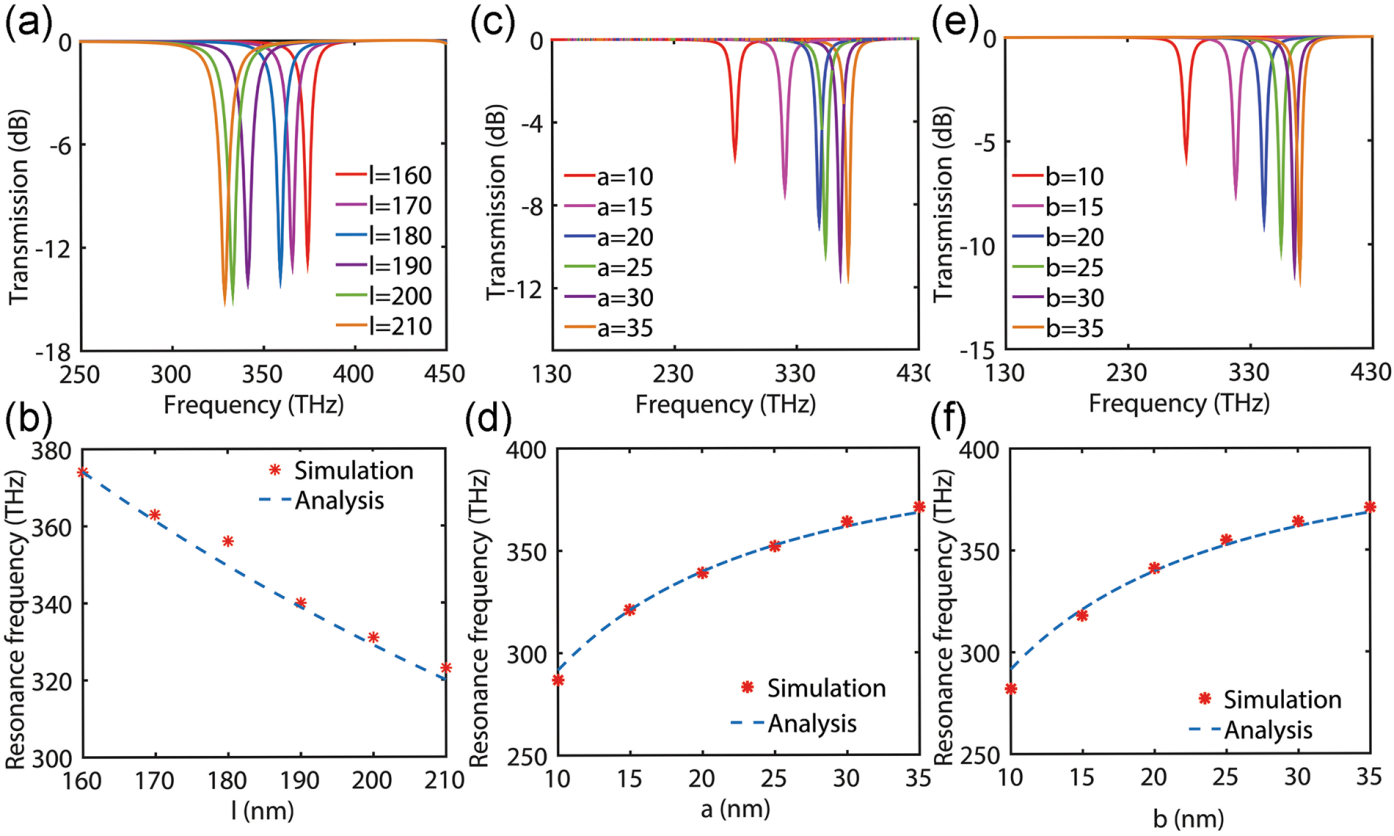
(**a**) Simulated transmission spectra and (**b**) resonance frequency of the rectangular plasmonic nanorod plot as a function of the height *l*. The length of the *a* and *b* are fixed as 30 nm and 40 nm, respectively. (**c**) Simulated transmission spectra and (**d**) resonance frequency of the rectangular plasmonic nanorod plot as a function of the width *a*. The length of the *l* and *b* are fixed as 160 nm and 30 nm, respectively. (**e**) Simulated transmission spectra and (**f**) resonance frequency of the rectangular plasmonic nanorod plot as a function of the width *b*. The length of the *l* and *a* are fixed as 160 nm and 30 nm, respectively. The stars represent the simulation data and the dashed line gives our analytic results.

As can be seen in Fig. 2, whether the parameter is the longitudinal length or lateral width of the rod, the results of the analysis and the simulation results are very consistent. For a perfectly conducting filament of negligible width (a & b → 0), the Faraday inductance can be neglected (*L*_*f*_ → 0), and the capacitance can be considered as a geometry-independent constant $(C\to \frac{a\pi {\varepsilon }_{b}}{2\,\mathrm{ln}(\sqrt{2}+1)})$. Thus, we obtain *f*_0_ ∝ (*a*/*l*)^1/2^, different with microwave theory *f*_0_ ∝ 1/*l*. On the other hand, for a thin rod in optical regime (*a* ≈ *b*, *a* & *b* ≪*l*), the Faraday inductance can also be neglected approximatively, because the mutual inductance calculations are no longer applicable to Faraday inductance calculations. As a result, the relationship *f*_0_ ∝ (*a*/*l*)^1/2^ can be obtained, similarly to ref.^42^ For the subwavelengh rectangular plasmonic nanorods, at optical frequencies the simple wavelength scaling breaks down because incident radiation is no longer perfectly reflected from a metal’s surface. Instead, radiation penetrates into the metallic nanorods and gives rise to oscillations of the free-electron gas. Moreover, it should be noted that, since the lateral parameters of the rectangular nanorod are smaller than the decay length (*a* & *b* < *δ*_*Ag*_) in the Fig. 2, the phase retardation can be neglected here.

### Rectangular Plasmonic Nanosheets of Sizes Comparable To the Wavelength

**Figure 3:**
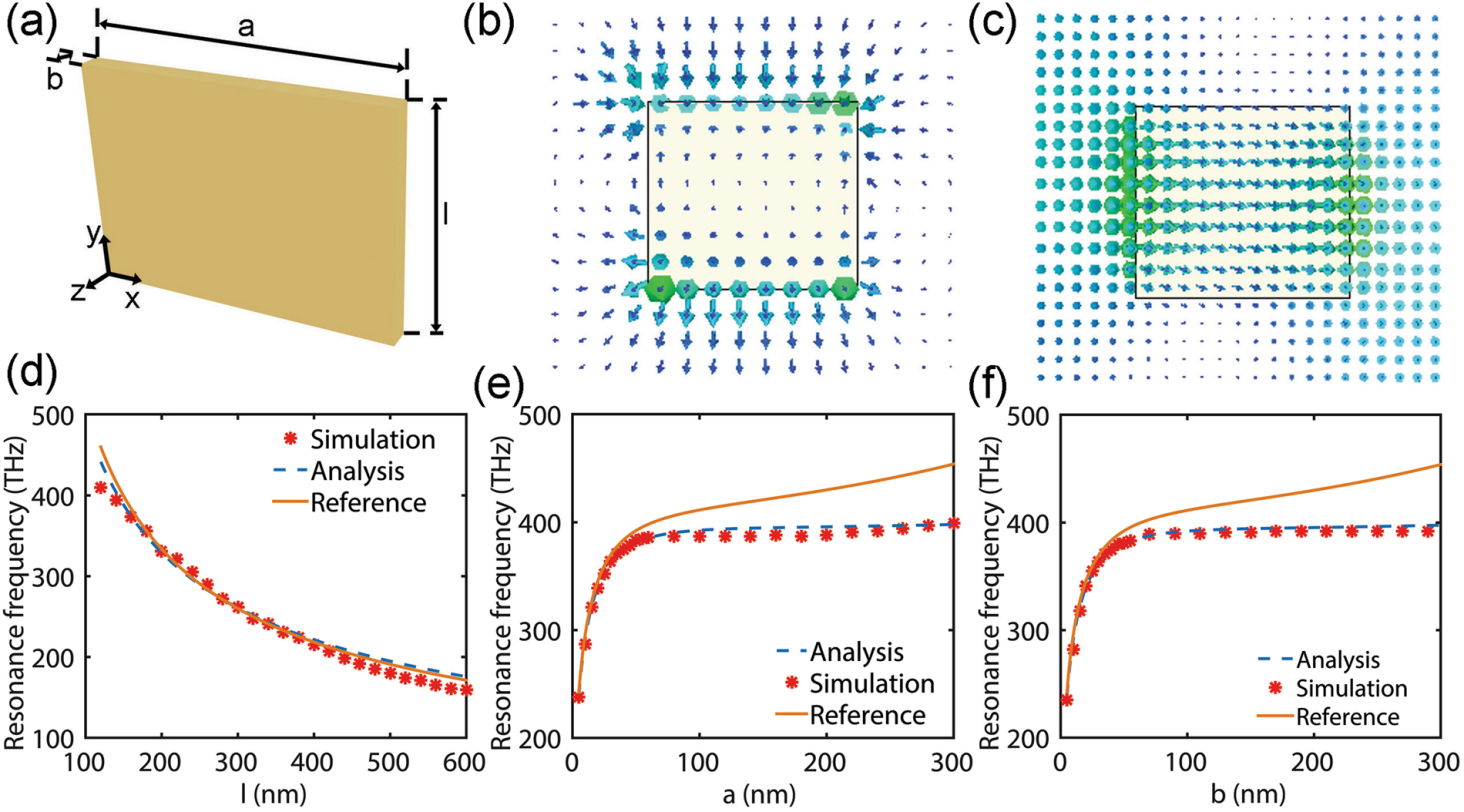
(**a**) Schematic of a plasmonic nanosheet embedded in air, and the incident light is propagating with the electric field along the rod axis, thus exciting the longitudinal plasmon resonance. (**b**) The electric field and (**c**) magnetic field distribution which located at z = -b/2 plane when a current flow in the rod is excited by the light electric field at 388 THz, *a* = 200 nm, *b* = 30 nm, *l* = 160 nm. Dependences of the resonance frequency on the (**d**) *l* [*a* = 30 nm, *b* = 40 nm], (**e**) *a* [*l* = 160 nm, *b* = 30 nm], and (**f**) *b* [*l* = 160 nm, *a* = 30 nm], respectively. Resonance frequencies extracted by the analytic eq. 4 compared against those extracted by the simulation (star *) and ref.^38^ (solid line).

But for the rectangular plasmonic resonators with the transverse characteristic dimensions larger than the decay length as shown in the Fig. 3a, the retardation will take its toll, because the spatial extension of the structure is not negligible in comparison to the wavelength. For visualizing the excited plasmonic resonance, the field distributions of the fundamental longitudinal resonances have been calculated for the present structure. In all simulations, the incident light propagates along the -z axis with the electric field along the y axis. Results for the distribution of the electric field and magnetic field are shown in Fig. 3b,c, respectively. Obviously, in the present nanosheet, the resonance frequency will be influenced more strongly by the inhomogeneous distribution of the fields which can be attributed to the phase retardation throughout the resonator. To further understand these effects, we compare the results from the master equation eq. 4 with the formula recently published in ref.^38^, in which the retardation effect was not considered. For the influence on the longitudinal length of the nanosheet, we can see from the Fig. 3d, the results from these equations are all in good agreement with the numerical simulation. But for the rectangular nanosheet with larger transverse length as shown in Fig. 3e,f (a or b > 50 nm) that, somewhat surprisingly, retardation is already important and shifts the resonance frequency to lower energies. In other words, the theory neglected retardation may be just suitable for describing the nanorod with large height-diameter aspect ratio.

## The Universality of the Analytical Expression

### Folded Rectangular Plasmonic Nanorods with Different Shapes

**Figure 4:**
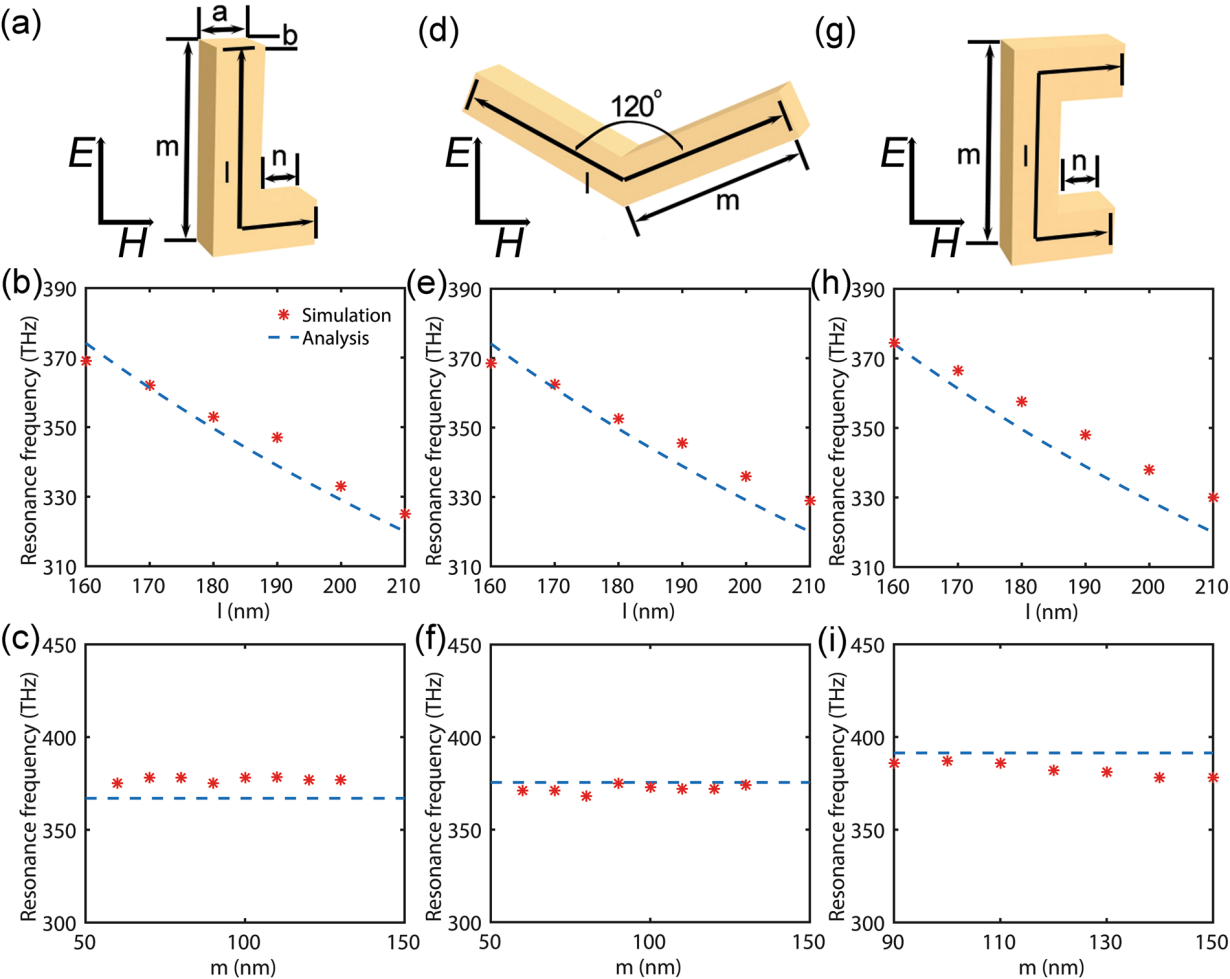
(**a**) Schematic of a L-shaped nanoresonator embedded in air, and the incident light is propagating with the electric field along the rod axis, thus exciting the longitudinal plasmon resonance. Dependences of the resonance frequency on the (**b**) different longitudinal total lengths *l* with *m* = 150 nm, and (**c)** arm lengths *m* of the L-shaped nanoresonator with *l* = 160 nm, respectively. Corresponding results of the other plasmonic resonators: [(**d**–**f**)] V-shaped nanoresonators and [(**g**–**i**)] the U-shaped nanoresonators. The lengths of the *a* and *b* are equal and fixed at 30 nm. And in the case of (**f**) and (**i**), the length of *l* is 160 nm. Resonance frequencies extracted by the analytic eq. 4 compared against those extracted by the simulation (star _*_).

To verify eq. 4, we numerically simulated the resonance frequency of folded rectangular silver nanorods with different shapes, namely L-, V- and U-shaped resonators. The simulated results are plotted in Fig. 4. Although L-shape is the noncentrosymmetric geometry which rod cannot reserve, the L-shaped resonators can still be considered as being folded from the rectangular nanorod. From a plasmonic point of view, the fundamental resonance is characterized by a charge oscillation along the entire rod that forms the L-shaped resonators. Indeed, like plasmon resonance in the rectangular nanorod, similar electromagnetic fields distribution can be observed in L-shaped resonators as well^43^. Thus, it is possible to exhibit similar resonance behaviors between the nanorods and the L-shaped nanoresonators with the same nominal total edge length. To test the accuracy of eq. 4 for these resonators with different longitudinal total lengths *l* and arm lengths *m*, we compare the resonance frequency *f*_0_ predicted with eq. 4 to the exact value obtained with the simulation. As plotted in Fig. 4, both theory and numerical experiments show that, the different arm lengths do not affect the resonance frequency at a certain total length. But an increasing total length results in a plasmonic resonance shift towards lower frequencies. In addition, the theoretical value predicted by eq. 4 is slightly lower than the simulation result. This phenomenon can be understood by unfolding conceptually the ‘L’ into an extended rod (the total length of a L shape is defined as the geometric mean of the long and short sides, i.e. $\sqrt{(m+a+n)(m-a+n)}$) that supports the so-called *LC*-resonance. Similar to the case of nanorods, due to the modified effective inductance and capacitance, the resonance frequency *f*_0_ will decrease when the rod length *l* increase as shown in Fig. 2d. And the V- and U- shaped resonators exhibit similar physics. Furthermore, it is possible that the model presented here can be extended to predict the resonant frequency of the polarization dependent on the incident. For instance, the doubling of the resonance frequency may be observed if the electric field polarization is chosen parallel to the legs of the structure as shown in Fig. 4(g). Because for this polarization, the internal field has to be in phase at both legs of the U-shaped resonator. The U-shaped resonator has mirror symmetry with respect to this polarization. For preserving this symmetry, the fields have to have an equal phase along the legs of the ‘U’-structure. Hence, the calculation of the effective inductance should be considered as parallel circuit and the length used to calculate a single inductor is approximately equal to half the effective length of the folded nanorods.

### Folded Rectangular Plasmonic Nanorods with Different Surrounding Mediums

**Figure 5:**
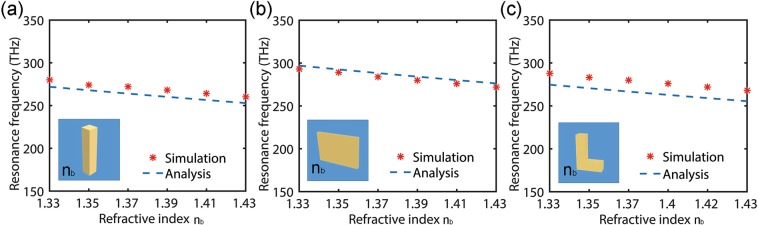
The resonance frequency changes against the refractive index of the surrounding medium for three plasmonic nanoresonators with different shapes, where the logitudinal length *l* is fixed as 160 nm and the shape is set as rod, sheet, and L-shape, respectively (from the left to the right): (**a**) a = 30 nm, b = 30 nm; (**b**) a = 180 nm, b = 30 nm; (**c**) a = 30 nm, b = 30 nm, m = 130 nm.

Moreover, the resonance peak position is very sensitive to the refractive index change of the surrounding medium of the plasmonic nanoresonators. A change in the refractive index of the surrounding environment results in a change in the effective capacitor of the *LC*-resonance presented here, which can be monitored by the shift of the resonance peak positions. To quantitatively prove that the expression is universally applicable to various plasmonic nanoresonators with different surrounding mediums, three different plasmonic nanoresonators were used in Fig. 5 for the comparison between the value predicted with eq. 4 and the numerical results. In general, the agreement between the analytical predictions obtained with eq. 4 and the fully vectorial calculations is excellent in Fig. 5a,b. Though there is a slight deviation in L-shaped resonator as shown in Fig. 5c, the theoretical peak-frequency shifts are still consistent with the numerical results (note that, the correction factors *α* and *β* used for L-shaped resonators and nanorods are the same). This unique property of plasmonic resonators can be used for sensing^33,34^. Moreover, we have performed additional tests on the plasmonic resonators composed of different folded rectangular geometries (i.e. nanorod, nanosheet, L-shaped nanoresonator, V-shaped nanoresonator with the 120° angle between the two arms, V-shaped nanoresonator with the 60° angle between the two arms, and the U-shaped nanoresonator) with different metallic constituents (that is Ag, Cu vs Al), and similar agreement was achieved as shown in Supporting Information IV. Although the resonance behaviors of those nanostructures exhibit a great diversity depending on the shape and material, clear relation between the resonance frequency and the shape, material, or surrounding medium can still be observed according to eq. 4.

## Summary

In this work, we derived a simple explicit expression to predict the fundamental resonance frequency of plasmonic nanoresonators with rectangular geometry. Interacted with the electromagnetic field localized in the resonators, the free electrons in the metal oscillate in harmonic way under the electromagnetic force, which generates the effective *LC*-resonance. Based on the classical electromagnetism, the effective kinetic inductance, Faraday inductance and capacitor of the plasmonic nanoresonators with rectangular geometry are explicitly described and supported by the numerical simulations. The geometric influences on the resonance frequency demonstrate that these novel expressions can be applied to different designs. This pure lumped circuit analogue of localized surface plasmon, without the aid of any numerical simulations, supplies an analytical approach for designing the resonance behaviors, which would open a wide range of possibilities and bring fantastic potential to plasmonic nanoresonators. We emphasize that the present approach is not stringently restricted by the subwavelenth limits and it may be used for nanoresonators of sizes comparable to the wavelength. Thus, it would be interesting to consider extension of the present work to coupled plasmonic systems that operate in flexible wavelength range, such as terahertz systems sustaining plasmonic resonances^44,45^.

## Supplementary information

Supporting Information
