# Supplementary material for: The Generalized Analytical Expression for the Resonance Frequencies of Plasmonic Nanoresonators Composed of Folded Rectangular Geometries
Source: Sci Rep. 2019 Jan 10;9:52. doi: 10.1038/s41598-018-37275-2 (PMC6328605; doi:10.1038/s41598-018-37275-2)
Supplement: Supplementary file 1 — Supporting Information [file 41598_2018_37275_MOESM1_ESM.doc]

Supporting Information

The Generalized Analytical Expression for the Resonance Frequencies of Plasmonic Nanoresonators Composed of Folded Rectangular Geometries

Hai Lu^1,2*^, Lijun Li^1^, Jun Zhang^1^, Shiqiang Xia^1^, Xiubao Kang^2^, Meng Huang ^1^, Kesheng Shen^1^, Chao Dong^1^, and Xianzhou Zhang^1^

*^1^Engineering Laboratory for Optoelectronic Technology and Advanced Manufacturing, Henan Normal University, Xinxiang 453007, China*

*^2^Key Laboratory of Advanced Micro-structured Materials, Ministry of Education, School of Physics Science and Engineering, Tongji University, Shanghai 200092, China*

*^*^E-mail:* [*luhai123@gmail.com*](mailto:luhai123@gmail.com)

**Content of the document**

I. Derivation of the kinetic inductance of plasmonic resonators with rectangular geometry

II. Derivation of the Faraday inductance of plasmonic resonators with rectangular geometry

III. Derivation of the capacitance of plasmonic resonators with rectangular geometry

IV. Universality of the Analytical Resonance Frequency Expression

**I. Derivation of the kinetic inductance of plasmonic resonators with rectangular geometry**

Consider a rectangular plasmonic rod of longitudinal length *l*, and its rectangular cross-sectional area *s* = *a* × *b.* The plasmon in this rod can be treated as a collective mechanical oscillation of the free electrons. Though it can be described as a quantum of plasma oscillation, this terminology is somewhat misleading as the phenomenon itself can be described accurately by classical physics. According to electrostatics, the displacement of the electron gas with respect to the background ionic lattice creates a built-in electric field *V*/*l*. Hence, the force on each electron can be written as:

$F=\frac{qV}{l}=\text{ }m_{e}\frac{dv}{dt}\text{ }=\text{ }m_{e}\frac{nqs}{nqs}\frac{dv}{dt}$ (1)

where *q* is the electric charge, *m_e_* is electron mass, *v* is the speed of the electrons driven by the external field, and *n* is the free-electron density in the metal. If we express the electric current *I* = *nqsv*, the eq 1 can be rewritten as:

$\frac{qV}{l}=\text{ }\frac{m_{e}}{nqs}\frac{dI}{dt}$ (2)

It means that a voltage response of an inductor can be obtained as:

$V=\text{ }\frac{m_{e}l}{nq^{2}s}\frac{dI}{dt}$ (3)

As a result, the kinetic inductor can be *L_k_* can be expressed as:

$L_{k}=\frac{m_{e}l}{nq^{2}s}=\frac{\mu lc^{2}}{\omega_{p}^{2}s}$ (4)

where *ω_p_* = (*nq*^2^/*mε*_0_)^1/2^ is the bulk plasma frequency, *s* = *a* × *b* is the cross-section area of rectangular nanorod, *ε_0_ and μ_0_* are permittivity and permeability of vaccum, and *c* is the light velocity, respectively.

**II. Derivation of the Faraday inductance of plasmonic resonators with rectangular geometry**

In order to simplify the consideration, we assume that the width of the cross-section is much smaller than the longitudinal length of the rectangular plasmonic nanorod. The Neumann formula for the mutual inductance of any two circuits is$M=\frac{\mu}{4\pi}\int_{i1} \int_{i2} \frac{dl_{1}dl_{2}}{D}\cos\theta$, where$D$is the distance between *dl*_1_ and *dl*_2_ in both circuits. Then, we can get the mutual inductance between two parallel wires:

$M=\frac{\mu}{4\pi}\int_{i1} \int_{i2} \frac{dl_{1}dl_{2}}{D}\cos\theta$ (5)

By sovle the integral in eq 5, we can get

$M=\frac{\mu l}{2\pi}\left(\ln\frac{\sqrt{l^{2}+d^{2}}+l}{d}-\frac{\sqrt{l^{2}+d^{2}}}{l}+\frac{d}{l}\right)$ (6)

Commonly, if exact values of inductance are not required, the equivalent self-inductance often may be calculated easier using geometric-mean-distances. It should be remembered, however, that even exact equations gives approximate values of inductance because most of these formulas assume that lengths are infinite. Thereby, the Faraday inductance can be expressed as:

$L_{f}=\frac{\mu l}{2\pi}\left(\ln\frac{\sqrt{l^{2}+g^{2}}+l}{g}-\frac{\sqrt{l^{2}+g^{2}}}{l}+\frac{g}{l}\right)$ (7)

where *g* = 0.2236(*a*+*b*) is the geometric mean distance of the cross section of the rectangular nanorod. In fact, the inductance of complicated geometries or of conductors having nonuniform current densities often can be calculated from a consideration of the magnetic energy of the conductor (*w*=*LI*^2^/2). For a thin rod in optical regime, since the internal part of the magnetic energy is much smaller than the external part, the Faraday inductance could be neglected in the calculation. In addition, when *a* ≈ *b*, *a* & *b* << *l*, the function $\ln(\sqrt{l^{2}+a^{2}}-l)$ approach to negative infinity, and it will be difficult to get the analytical solution of the eq 5.

**III. Derivation of the capacitance of plasmonic resonators with rectangular geometry**

As stated above, as a consequence of the current flow, electric charges with different signs ±*q* will accumulate on the opposite end-caps of the nanorod. The charge distribution σ creates an oscillatory voltage *V* which can be calculated from electrostatics. With voltage and charge calculated, an effective capacitance can be determined. Firstly, consider a homogeneous two dimensional charge distribution in the plane, and then the potential at the center of the rectangular end-cap can be expressed as:

$\begin{matrix} V=\int_{-a/2}^{a/2} \int_{-b/2}^{b/2} \frac{\sigma dzdy}{4\pi\varepsilon_{b}\sqrt{y^{2}+z^{2}}} \\ =\frac{q}{ab\pi\varepsilon_{b}}\left[\frac{b}{2}\ln\frac{\sqrt{b^{2}+a^{2}}+a}{b}-\frac{a}{2}\ln\left|\frac{b-\sqrt{b^{2}+a^{2}}-a}{b+\sqrt{b^{2}+a^{2}}+a}\right|\right] \end{matrix}$ (8)

where *a* and *b* are the transverse geometrical parameters of plasmonic nanorod, and *ε*_b_ is the permittivity of the background. Based on eq 8, we can get the potential of a rectangular plate with a positive charge at its center:

$V_{+}=\frac{q}{ab\pi\varepsilon}\left[\frac{b}{2}\ln\frac{\sqrt{b^{2}+a^{2}}+a}{b}-\frac{a}{2}\ln\left|\frac{b-\sqrt{b^{2}+a^{2}}-a}{b+\sqrt{b^{2}+a^{2}}+a}\right|\right]$ (9)

On the other hand, the potential at the center of the rectangular plate with uniformly negative charged is

$V_{-}=\frac{-q}{ab\pi\varepsilon}\left[\frac{b}{2}\ln\frac{\sqrt{b^{2}+a^{2}}+a}{b}-\frac{a}{2}\ln\left|\frac{b-\sqrt{b^{2}+a^{2}}-a}{b+\sqrt{b^{2}+a^{2}}+a}\right|\right]$ (10)

According to equations (9) and equation (10), we can work out anaiytically with the potential difference between the two disk centers

$U=V_{+}-V_{-}=\frac{q}{ab\pi\varepsilon}\left[b\ln\frac{\sqrt{b^{2}+a^{2}}+a}{b}-a\ln\left|\frac{b-\sqrt{b^{2}+a^{2}}-a}{b+\sqrt{b^{2}+a^{2}}+a}\right|\right]$ (11)

If the capacitance *C* is defined simply as the electric charges divided by the potential difference between the two disk centers, we have

$C=\frac{ab\pi\varepsilon_{b}}{b\ln\frac{\sqrt{a^{2}+b^{2}}+a}{b}+a\ln\frac{b+\sqrt{a^{2}+b^{2}}}{a}}$ (12)

It should be mentioned that the charge distribution of the rectangular disk is not uniform in reality, so the necessary amendments are needed for the effective capacitance. Moreover, similar correction processing is also applicable to the inductance calculation due to the internal magnetic energy that cannot be ignored.

**IV. Universality of the Analytical Resonance Frequency Expression**

To verify eq 4 in the main text, we consider six types of plasmonic nanoresonators composed of different rectangular geometries with different metallic constituents (i.e. Ag, Cu, and Al): nanorod, nanosheet, L-shaped nanoresonator, V-shaped nanoresonator with the 120° angle between the two arms, V-shaped nanoresonator with the 60° angle between the two arms, and the U-shaped nanoresonator. Comparison between the theoretical results and simulation results are shown in Figure 1. For different shapes of plasmon nanoresonators made by the same constituent, their correction factors are consistent. Here, the correction factors *α*(*β*) are chosen as 2.65(0.65), 2.6(0.55) and 4.1(0.55) for silver, copper and aluminum, respectively. It should be noted that, inhomogeneous distribution of charges on the metal surfaces is not the only cause of correction factors. For an effective *LC* circuit, the electrostatic energy, the electron kinetic energy and the total magnetic energy should be stored in *C*, *L_k_* and *L_f_*, respectively. Taking into account the scale of the nanoresonator and the high frequency drive field at optical regime, the electron kinetic energy should be much larger than that of magnetic energy and electrostatic energy. As shown in eq 1 in the main text, the bulk plasma frequency *ω_p_* dominates this term. Thus, it looks like the correction factor seems to depend only on the material. In addition, this demonstrates that the model proposed in this paper can not be applied to low frequency range in which the magnetic energy and electrostatic energy may play different roles. In the simulation, realistic parameters of Cu and Al were chosen to be *ω_p_*_(Cu)_ = 6.38 × 10^4^ cm^-1^, Γ_(Cu)_ = 2.78 × 10^2^ cm^-1^, *ω_p_*_(Al)_ = 1.19 × 10^5^ cm^-1^, and Γ_(Al)_ = 6.47 × 10^2^ cm^-1^, respectively.^1^

As can be seen in Figure 1, from nanorods to U-shaped resonators, the agreement between the analytical predictions obtained with eq 4 in the main text and the numerical simulations is excellent for different metals. It is worth mentioning that, for the occurrence of a larger deviation in V-shaped nanoresonator with the 60° angle between the two arms as shown in Figure 1e, at least two physical interpretations can be given. The first interpretation is that, in electrical engineering terms, the effective capacity may be modified by the additional capacity resulting from the two near end faces of the nanorod. In the second interpretation, from a plasmonic point of view, the longitudinal resonance in this polarization is dominantly associated with a plasmon excitation in the two arms of the V-shaped structure. The extreme folding reduces the effective length *l* of the nanorods and shifts the resonance to higher frequencies.

**REFERENCES :**

(1) Ordal, M. A.; Long, L. L.; Bell, R. J.; Bell, S. E.; Bell, R. R.; Alexander, R. W.; Ward, C. A. Appl. Opt. 1983, 22(7), 1099-1119.


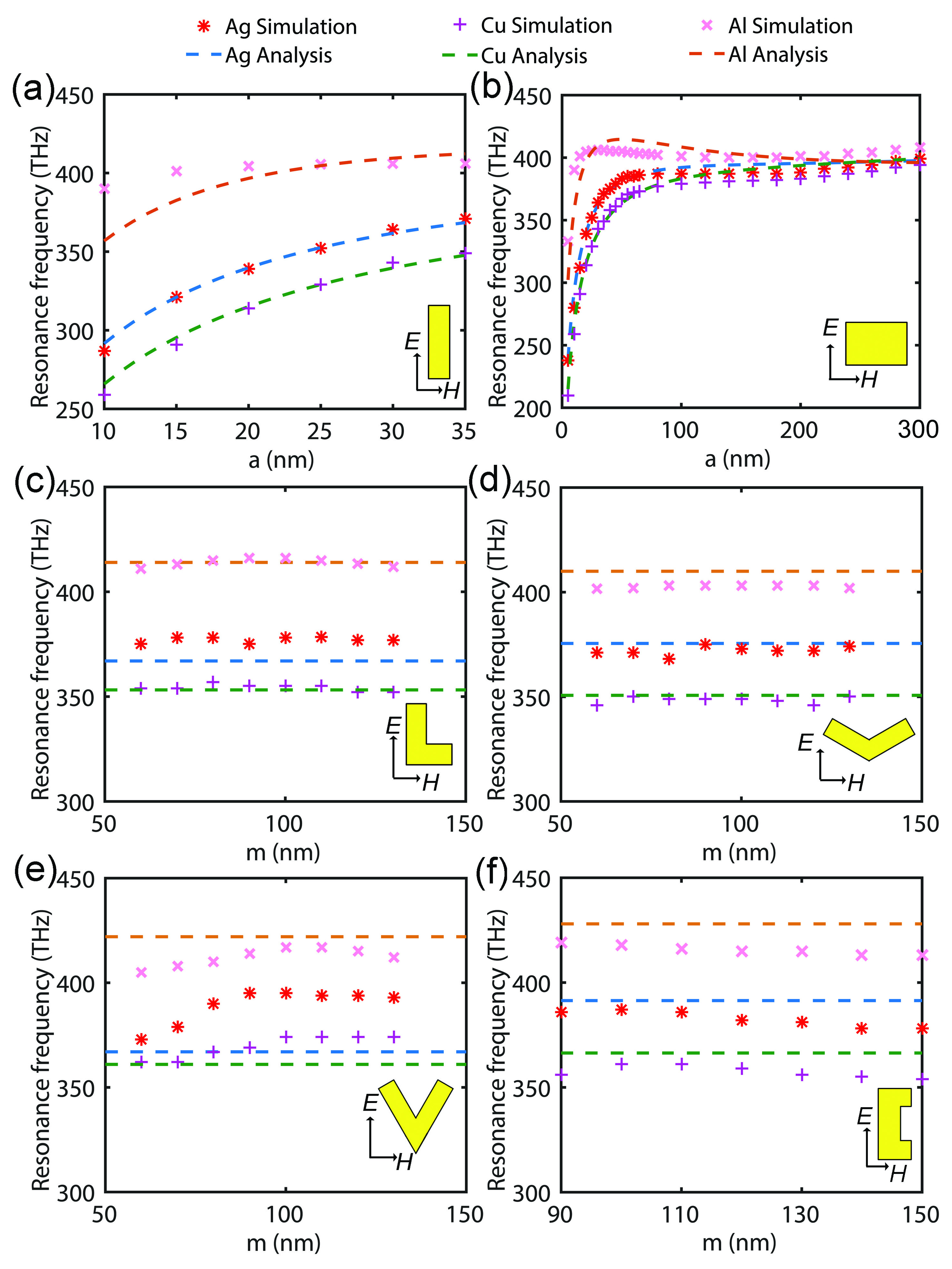


**Figure** 1. Resonance frequency as a function of the geometical parameters for rectangular plasmonic nanoresonators with different metallic constituents. Simulation and analytic results are plotted as symbols, and dashed lines, respectively, for six different types of plasmonic resonators: (a) a nanorod and (b) nanosheet with different width *a*, where *b* = 30nm, *l* = 160nm. (c) L-shaped nanorods with different truncation length *m* (namely, the length of the arms). (d) and (e) are V-shaped resonators with different arm lengths. And the angle between the two arms are the 120° and 60°, respectively, where a = *b* = 30nm, *l* = 160nm. (f) U-shaped resonators with different arm lengths, where a = *b* = 30nm, and *l* = 160nm.
